# Costs, effects and implementation of routine data emergency admission risk prediction models in primary care for patients with, or at risk of, chronic conditions: a systematic review protocol

**DOI:** 10.1136/bmjopen-2015-009653

**Published:** 2016-03-01

**Authors:** Mark Rhys Kingston, Bridie Angela Evans, Kayleigh Nelson, Hayley Hutchings, Ian Russell, Helen Snooks

**Affiliations:** Swansea University Medical School, Swansea, UK

**Keywords:** PRIMARY CARE, Risk management < HEALTH SERVICES ADMINISTRATION & MANAGEMENT, PREVENTIVE MEDICINE, EPIDEMIOLOGY

## Abstract

**Introduction:**

Emergency admission risk prediction models are increasingly used to identify patients, typically with one or more chronic conditions, for proactive management in primary care to avoid admissions, save costs and improve patient experience.

**Aim:**

To identify and review the published evidence on the costs, effects and implementation of emergency admission risk prediction models in primary care for patients with, or at risk of, chronic conditions.

**Methods:**

We shall search for studies of healthcare interventions using routine data-generated emergency admission risk models. We shall report: the effects on emergency admissions and health costs; clinician and patient views; and implementation findings. We shall search ASSIA, CINAHL, the Cochrane Library, HMIC, ISI Web of Science, MEDLINE and Scopus from 2005, review references in and citations of included articles, search key journals and contact experts. Study selection, data extraction and quality assessment will be performed by two independent reviewers.

**Ethics and dissemination:**

No ethical permissions are required for this study using published data. Findings will be disseminated widely, including publication in a peer-reviewed journal and through conferences in primary and emergency care and chronic conditions. We judge our results will help a wide audience including primary care practitioners and commissioners, and policymakers.

**Trial registration number:**

CRD42015016874; Pre-results.

**Strengths and limitations of this study**

- The strength of this review is its extensive, unbiased search of multiple databases, journals, references and citations, combined with consulting experts. The review is broad to ensure a thorough understanding of evidence relating to the impact of emergency admission risk models from a range of study designs.
- Limitations are that our review does not include grey literature (as we are focusing on peer-reviewed literature), and that study heterogeneity may present challenges for data synthesis.
- The results of this systematic review will help strengthen the evidence base and inform the debate over best practice for managing patients at risk of emergency admission.

## Introduction

An ageing population and rising incidence of chronic conditions places unprecedented demand on healthcare services.^1^ ^2^ Patients with chronic conditions are more likely to experience emergency hospital admissions for potentially avoidable causes resulting in suboptimal health outcomes, increased health costs and poor patient experience.^3^ Primary and community care can deliver efficient, coordinated but individualised care that can prevent emergency admissions, reduce costs and improve care quality.^4^ However, preventive interventions must be targeted at those genuinely at risk if they are to be effective.^5–7^

Emergency admission predictive risk models have been widely developed in response to the growing international burden of disease. They use mathematical formulae to interpret patient-level data (eg, age, previous health service use and diagnosed chronic conditions) to identify those at risk of emergency admission. A recent systematic review of the technical performance of emergency admission risk models identified 27 validated models for use in primary care and considerable international research into their development and validation.^8^ The authors concluded that models using routinely collected clinical patient data performed well—and better than those requiring self-reported patient data. Furthermore, models reliant on self-reported (questionnaire) data are limited by response rates, recall issues and respondent burden.^9^

Other approaches to patient selection for interventions targeting high-risk patients, such as simple referral criteria—known as ‘threshold modelling’—or identification by clinicians unaided by a risk model, have been largely discredited owing to poor predictive accuracy,^5^ ^7^ ^10^ and are therefore outside the scope of this review.

Emergency admission risk prediction models are widely advocated in international policy and practice, most notably in the UK, Europe and USA.^11–14^ England recently introduced a primary care-enhanced service with funding of £160 million per annum to encourage general practitioners to use models to help identify high-risk patients for active management in the community.^15^ Despite these developments, there is no clear consensus on the best interventions for identified patients, which patients to target or what effects to expect.^3^ ^6^

In this systematic review, we aim to examine the effects, costs and implementation of using risk prediction models in primary care to identify patients with chronic conditions at risk of future emergency admissions. To our knowledge, no review has been undertaken into the effectiveness of these risk prediction models. Given international interest and policy focus on these models, this review is needed to identify and review published studies, strengthen the evidence base and inform best practice in managing patients at risk of emergency admission.

## Methods

We registered this systematic review with PROSPERO—the International Prospective Register Of Systematic Reviews on 14 April 2015 (reference: CRD42015016874). The protocol conforms to the Preferred Reporting Items for Systematic review and Meta-Analysis for Protocols (PRISMA-P) guidelines,^16^ and the review will conform with the related PRISMA guidelines.^17^

### Research question

What are the effects, costs and facilitators of, and barriers to, implementing emergency admission risk prediction models in primary care for patients with, or at risk of, chronic conditions?

### Eligibility criteria

In addressing our research question, we shall include peer-reviewed studies published since 2005, with no language restrictions, that meet the criteria in table 1.

**Table 1:** Eligibility criteria

Population	Patients registered with general practices, or consulting primary or community care practitioners.
Intervention	Models in primary care using routine data to predict risk of hospital admission for patients with, or at risk of, chronic conditions. *Exclusion*: models that rely on patient-reported data.
Comparators	External (eg, trial) or internal (eg, cohort). None for qualitative studies.
Outcomes	Clinical or cost-effectiveness, views of patients or health professionals on emergency admission risk prediction models, or implementation of model.
Study design	Studies that report empirical data. *Exclusion*: commentaries or editorials.

### Search strategy

We shall carry out a comprehensive electronic search in ASSIA (via ProQuest), CINAHL (via EBSCO), the Cochrane Library (Cochrane Central Register of Controlled Trials and Economic Evaluations), HMIC (via Ovid), ISI Web of Science, MEDLINE (via EBSCO) and Scopus. We shall develop and pilot a search strategy with the support of a specialist librarian in an iterative process using the clinical prediction and prognostic search filters outlined by Ingui and Rogers^18^ and Geersing *et al*.^19^ Online supplementary appendix 1 lists the resulting search strategy for MEDLINE.

10.1136/bmjopen-2015-009653.supp1 Supplementary appendix

Once the MEDLINE search strategy has been reviewed and finalised, we shall adjust and develop it for our other data sources. We selected 2005 as the earliest publication date to precede relevant policy initiatives that prompted risk model development using routine data,^11^ ^12^ ^20^ ^21^ and to ensure relevance to contemporary primary and community care.

We shall also: hand search three journals known to have published in this field—*BMC Family Practice*, the *British Journal of General Practice*, and the *International Journal of Integrated Care*; use ISI Web of Science and Scopus to search the references and citations of included articles; undertake a secondary search using the names (or other identifiers) of risk models used in included articles; and consult experts in emergency admission risk prediction.

### Study selection

We shall use EndNote reference software to collate search results and remove duplicates. Two reviewers (MRK and KN) will independently assess initial eligibility of identified studies by screening titles, abstracts and keywords. KN will then obtain potentially eligible full texts for definitive assessment against the inclusion and exclusion criteria by MRK and BAE. Disagreements will be resolved through discussion. We shall record reasons for excluding full-text articles, and summarise the study selection process in a PRISMA flow diagram.^17^

### Data extraction

We shall develop a data extraction form that reflects the review aims and conforms with guidance from the National Health Service (NHS) Centre for Reviews and Dissemination.^22^ We shall pilot this form on a sample of studies and adjust as necessary. Two reviewers (MRK and HH) will extract data independently and in duplicate from all eligible studies. They will resolve differences through discussion with a third reviewer (BAE). Our primary outcome for data extraction shall be the number of emergency hospital admissions, given the risk models are typically advocated for use within emergency admission avoidance interventions. We will also extract data, where available, on:

- Details of the risk model used and of validation of the model;
- Use of non-emergency healthcare resources, implementation costs, reported facilitators and barriers, and clinicians’ and patients’ views, notably satisfaction;
- Study characteristics (setting, objectives, design and methods);
- Study population, notably selection criteria;
- Nature and purpose of risk prediction model; and
- Implementation of risk prediction model—who used it and how.

Where study data are unclear, we shall try to contact the corresponding author of the paper for clarification.

### Quality assessment

As we expect to include randomised and non-randomised studies, we shall use a quality and bias assessment tool^23^ designed to cover a range of quantitative research designs. This tool assesses study design, sample selection, identification and analysis of confounders, blinding of outcome assessors and participants, validity and reliability of data collection methods, and nature and extent of withdrawals. We shall also use the Walsh and Downe^24^ framework to appraise qualitative studies according to their scope and purpose, design, sampling, analysis, interpretation, reflexivity, ethical dimensions and relevance.

Two reviewers (MRK and HH) will independently assess general study quality as strong, moderate or weak. They will resolve differences through discussion with a third reviewer (BAE).

### Data synthesis

We shall tabulate the characteristics of included studies, including narrative summaries of risk prediction models, how they were implemented and their effects. If feasible, we shall analyse outcomes relating to clinical and cost-effectiveness by metaregression. We shall assess study heterogeneity by two statistics—Q based on the χ^2^ test and I^2^, which assesses how much variance is attributable to heterogeneity.^25^ We shall present quantitative data in forest and funnel plots when appropriate. Missing data from included articles will be sought through author contact. The impact of missing data will be discussed.

We shall use narrative synthesis to review qualitative data on model implementation, patients’ and clinicians’ views, and cost and clinical data too heterogeneous for meta-analysis through the four-step framework developed by Popay *et al*:^26^

- Developing a theory of how the intervention works, why and for whom;
- Generating a preliminary synthesis of findings of included studies;
- Exploring relationships in the data; and
- Assessing the robustness of the synthesis.

### Dissemination

We shall report our findings through peer-reviewed publication and conferences—in primary care, emergency care and chronic conditions. We shall consult representatives of patients, primary care, health policy and commissioners to tailor outputs to these audiences.

### Ethics

As this study uses published data, ethical permission is not necessary. Nevertheless, we shall set high ethical and governance standards in managing our data and presenting findings.

## Conclusion

The effectiveness of emergency admission risk prediction models is poorly understood. Although it is not clear whether their use influences patient outcomes or resource use, healthcare agencies are expected to use the models to allocate limited resources.^6^ This review will therefore identify and synthesise current evidence, covering: clinical and cost-effectiveness; how models have been used for patients with, or at risk of, chronic conditions; and barriers to, and facilitators of, their use. Results will inform decisions by healthcare commissioners on the future use of risk prediction models, and identify priorities for further research in this increasingly important field.
