# Supplementary material for: Costs, effects and implementation of routine data emergency admission risk prediction models in primary care for patients with, or at risk of, chronic conditions: a systematic review protocol
Source: BMJ Open. 2016 Mar 1;6(3):e009653. doi: 10.1136/bmjopen-2015-009653 (PMC4785313; doi:10.1136/bmjopen-2015-009653)
Supplement: Supplementary appendix [file bmjopen-2015-009653supp.pdf]

Appendix 1: Search strategy for MEDLINE (via EBSCO)

S1 predict* OR clinical* OR outcome* OR risk*

S2 (Predict* AND (Outcome* OR Risk* OR Model*)) OR ((History OR Variable* OR Criteria OR Scor* OR Characteristic* OR Finding* OR Factor*) AND (Predict* OR Model* OR Decision* OR Identif* OR Prognos*)) OR (Decision* AND (Model* OR Clinical* OR (MH "Logistic Models"))) OR (Prognostic AND (History OR Variable* OR Criteria OR Scor* OR Characteristic* OR Finding* OR Factor* OR Model*)) OR ((TI predict*) OR validat* OR rule*)

S3 (MH "ROC Curve") OR (‘‘Stratification’’ OR ‘‘Discrimination’’ OR ‘‘Discriminate’’ OR ‘‘c-statistic’’ OR ‘‘c statistic’’ OR ‘‘Area under the curve’’ OR ‘‘AUC’’ OR ‘‘Calibration’’ OR ‘‘Indices’’ OR ‘‘Algorithm’’ OR ‘‘Multivariable’’)

S4 (MH "Predictive Value of Tests") OR TI PREDICT* OR AB PREDICT*

S5 AB (SCOR* OR OBSERV*) OR TI (SCOR* OR OBSERV*) OR MH Observer Variation

S6 (S1 - S5/OR)

S7 “primary care” OR “primary health*” OR GP OR general practi* OR PCT* OR “health authorit*” OR community OR CCG* OR “family practi*” OR NHS OR "case management" OR "managed care" OR "integrated care" OR(MH "General Practitioners") OR (MH "Physicians, Family") OR (MH "Physicians, Primary Care") OR (MH "Primary Health Care") OR (MH "Primary Care Nursing") OR (MH "Community Health Services") OR (MH "Community Health Centers") OR (MH "Community Health Nursing") OR (MH "Primary Prevention") OR (MH "Disease Management+") OR "disease management"

S8 (rehospitali?ation* OR hospitali?ation* OR readmission* OR re-admission* OR admission*) OR “emergency referral*” OR (MH "Emergencies") OR (MH "Emergency Service, Hospital/TD/SN") OR (MH "Emergency Treatment+/TD/UT") OR (MH "Emergency Medicine") OR (MH "Emergency Medical Services+") OR (MH "Length of Stay/EC/SN/TD") OR overnight stay

S9 (S6 AND S7 AND S8)

S10 "chronic condition*" OR "chronic* ill*" OR "chronic disease*" OR "chronic care" OR ("LONG TERM" N1 (condition* OR ill* OR disease*)) OR (chronic N2 (condition* OR ill* OR disease*)) OR multimorbidity OR comorbidity OR multi-morbidity OR co-morbidity OR (MH “Chronic Disease+”)

S11 (S9 AND S10)
